# Supplementary material for: Cis-regulatory architecture downstream of FLOWERING LOCUS T underlies quantitative control of flowering in Arabidopsis thaliana
Source: Genome Biol. 2026 Apr 6;27:124. doi: 10.1186/s13059-026-04064-4 (PMC13067676; doi:10.1186/s13059-026-04064-4)
Supplement: Supplementary file 1 — Additional file 1: Supplemental Figures. Fig S1. Spatiotemporal expression pattern of the non-coding gene AT1G08757. Fig. S2. Full complementation of the late-flowering ft-10 phenotype by FT with upstream and downstream regulatory elements. Fig. S3. Conservation of cis-regulatory DNA motifs within Block E across Brassicaceae species. Fig. S4. Neither the upstream G-box nor the Block E G-box alone restores FT expression without the CCAAT-box. Fig. S5. MOA-seq reveals selective CCAAT-box occupancy changes around Block E in deletion mutants. [file 13059_2026_4064_MOESM1_ESM.pdf]

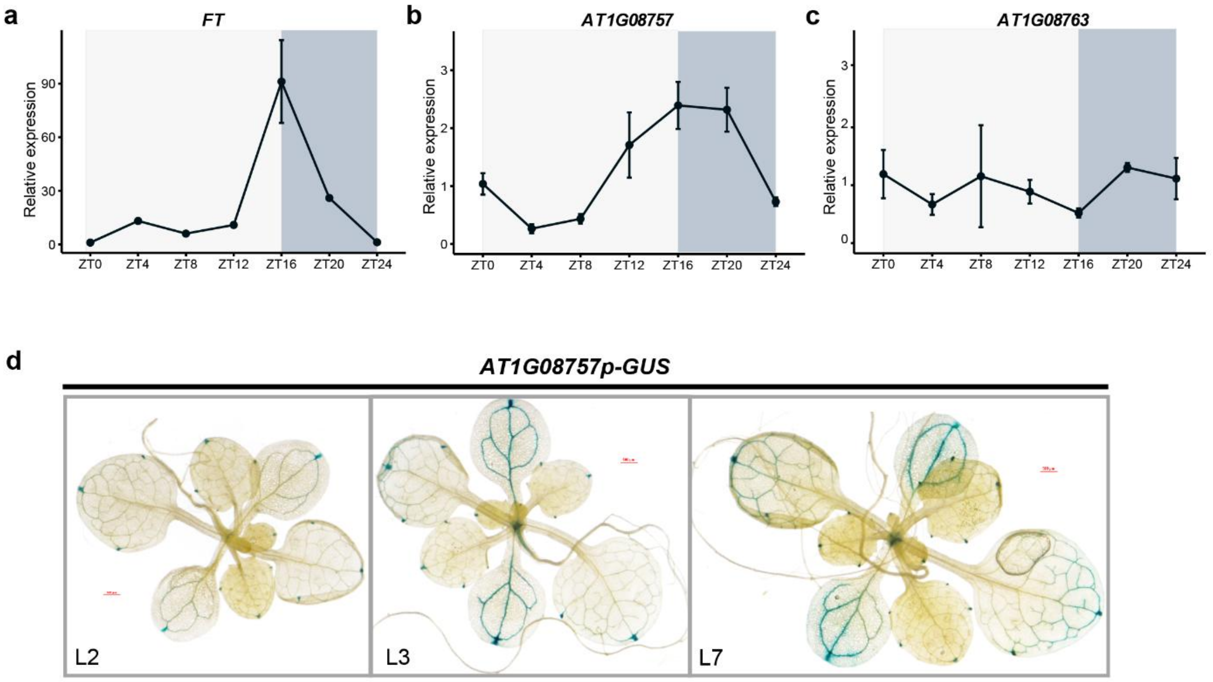

Fig. S1. Spatiotemporal expression pattern of the non-coding gene *AT1G08757*. **a–c**, Transcript accumulation measured by one step RT-qPCR from 14-day old seedlings grown under long-day (LD) at 21°C condition. Samples were collected in 4h intervals over a time-period of 24h. Error bars show standard error of the mean of three biological replicates. (**a**) *FT*, (**b**) non-coding transcript *AT1G08757*, (**c**) non-coding transcript *AT1G08763*. **d**, GUS staining of three independent *AT1G08757p-GUS* transgenic lines(L) grown under LD conditions. Scale bar: 500 µm.

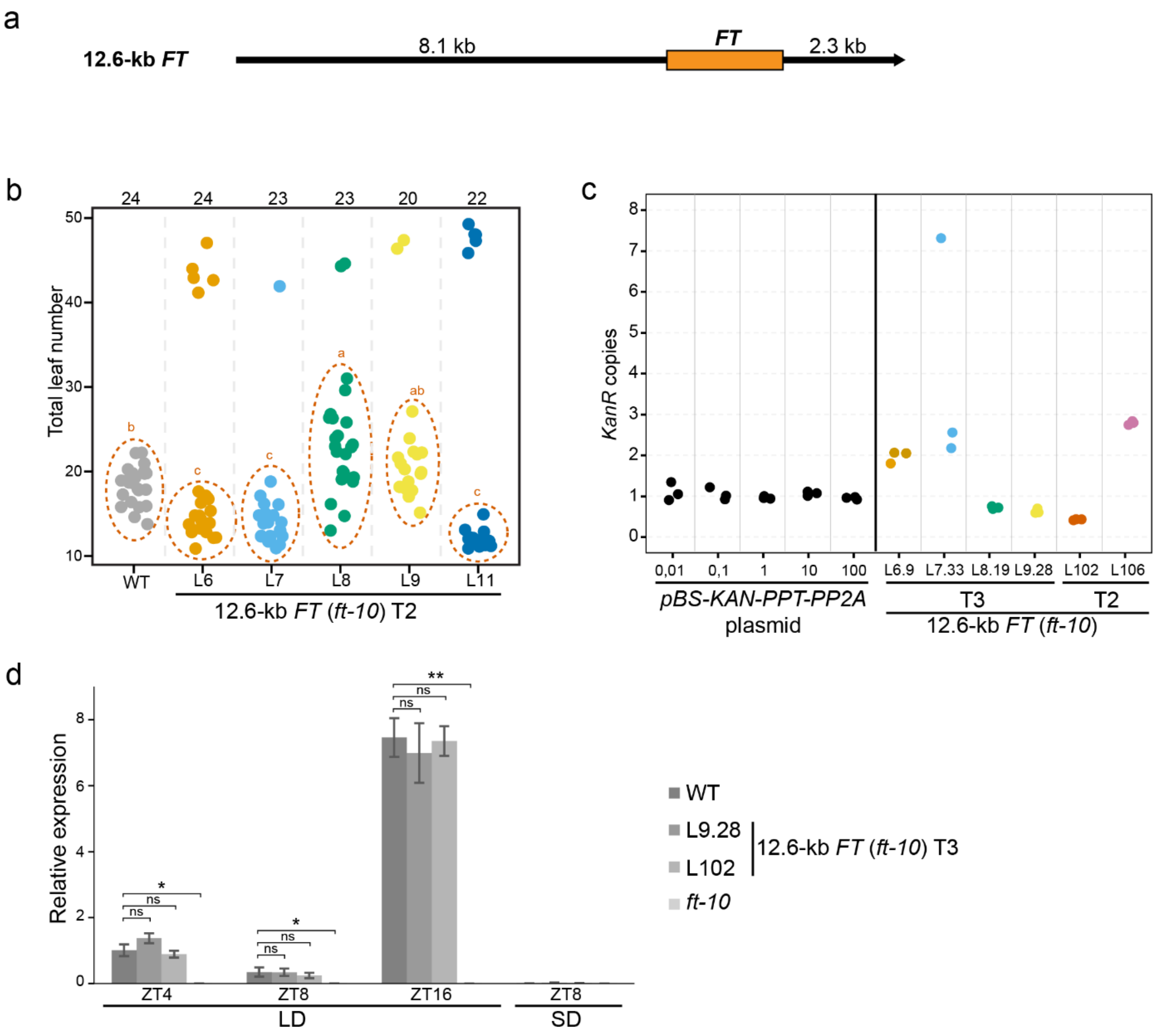

Fig. S2. Full complementation of the late-flowering *ft-10* phenotype by *FT* with upstream and downstream regulatory elements. a, Schematic representation of *FT* and its flanking sequences retrieved from BAC clone F5I14 by recombineering. The 12.6-kb *FT* genomic fragment (Chr1:24323415–24335986) spans 8.1 kb upstream of the start codon through 2.3 kb downstream of the stop codon, encompassing all known regulatory elements. **b**, Flowering time, measured as the number of leaves at bolting, in wild type (WT) and T2 populations of five independent transgenic lines carrying the 12.6-Kb *FT* genomic region. Replicate numbers are shown above the plot. For each line, plants flowering similarly to WT (dashed circles) were analyzed with WT (also circled) by ANOVA with Tukey's HSD. Different letters indicate significant differences.

c, Copy-number test of transgenic lines by qPCR. The ratio of *KanR*/*PP2A* determined from a reference plasmid (left panel) was compared to values obtained from T3 offspring of lines shown in **b** and from T2 plants of two additional lines. Values show technical replicates of qPCR results. Lines L8, L9 and L102 were evaluated as single copy, note that lines L7, L102, and L106 were not homozygous. **d**, *FT* transcript accumulation in WT, *ft-10*, and two independent single-copy transgenic lines, measured by RT–qPCR. Samples were collected at ZT4, ZT8, and ZT16 under long-day (LD) conditions and at ZT8 under short-day (SD) conditions. Relative expression was calculated using the 2^ΔΔCq method with *PP2A* as reference. Significance by Welch's t-test (ns $p>0.05$, * $p<0.05$, ** $p<0.01$, *** $p<0.001$).

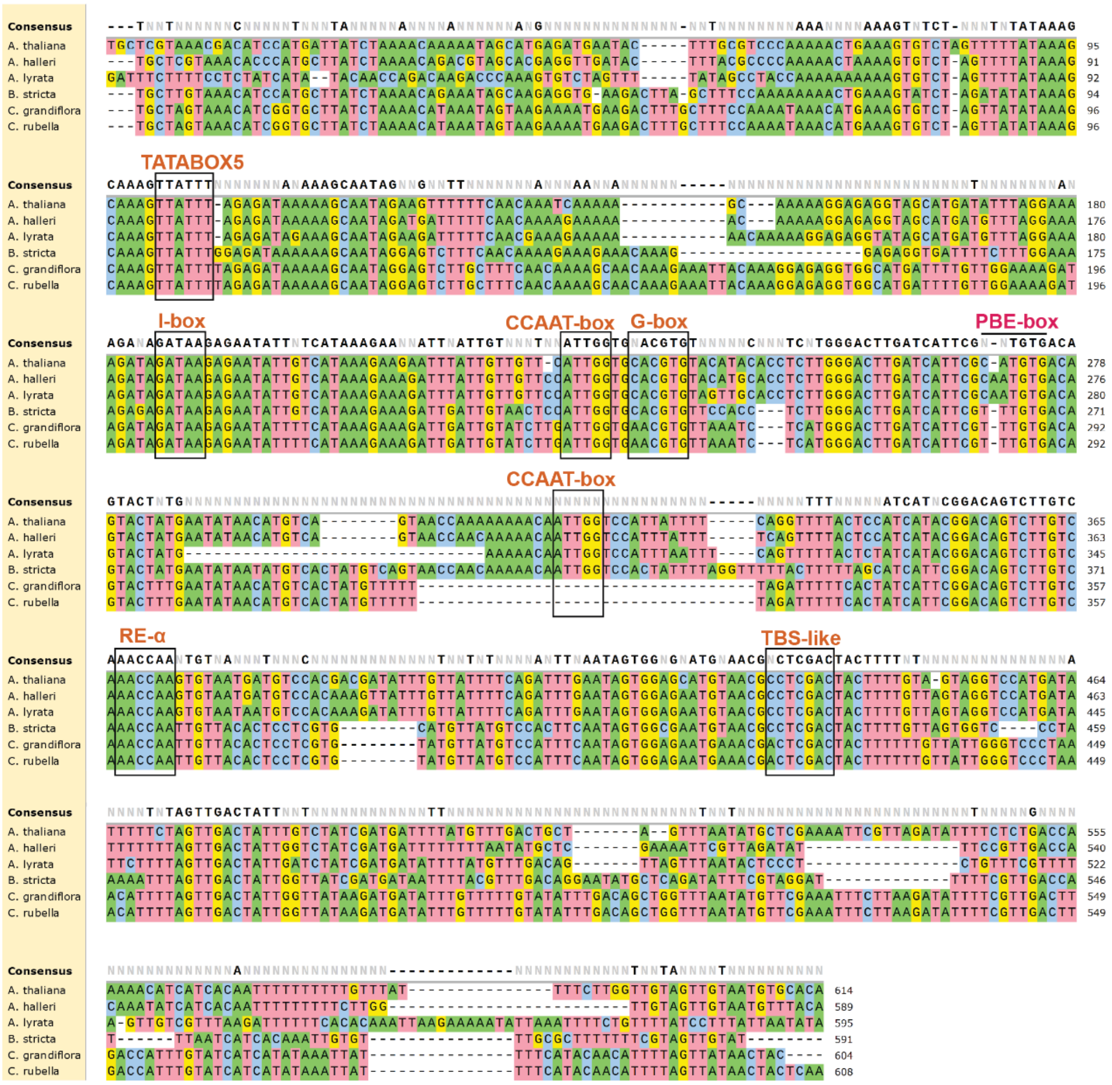

Fig. S3. Conservation of *cis*-regulatory DNA motifs within Block E across Brassicaceae species. The motifs TATABOX5, I-box, CCAAT-box, G-box, PBE-box, and RE-alpha were identified within *A. thaliana* Block E (Chr1:24,335,009–24,335,622) using the New PLACE database (https://www.dna.affrc.go.jp/PLACE/?action=newplace) [1]. The TBS-like motif, also known as MA0553.1, was previously described [2, 3]. Sequence alignment of Block E was performed using Clustal Omega in SnapGene across six related species: *A. thaliana*, *A. halleri*, *A. lyrata*, *B. stricta*, *C. grandiflora*, and *C. rubella*. Conserved occurrences of the motifs are highlighted with black boxes.

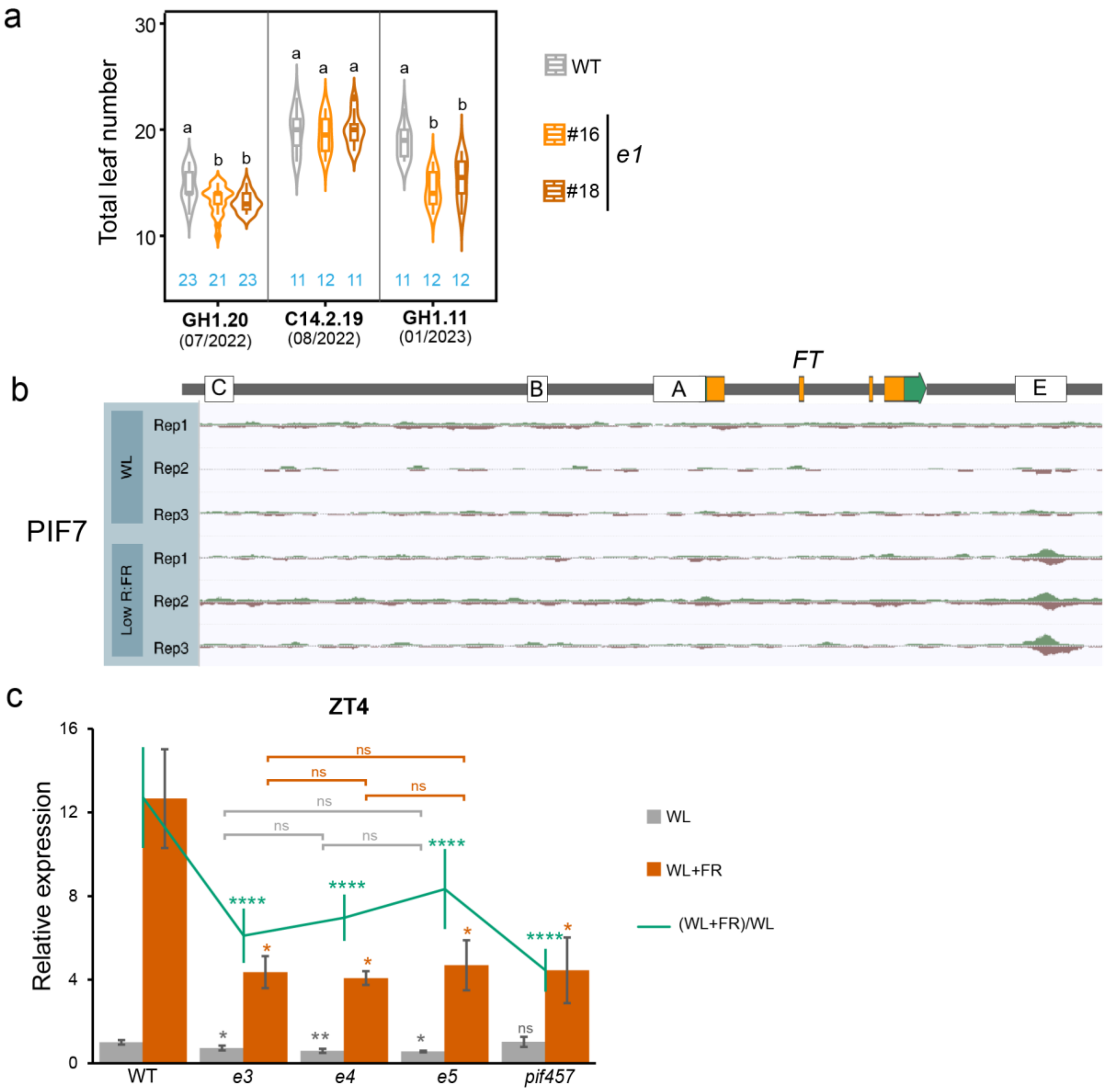

Fig. S4. Neither the upstream G-box nor the Block E G-box alone restores *FT* expression without the CCAAT-box. a, Flowering time measured as leaves produced at the main shoot until flowering in wild type (WT) and two independent segregants of a deletion after the second G-box downstream of *FT* (*e1*). Plants were grown in either greenhouse (GH) or growth chamber (C) conditions. Blue numbers within the plot indicate the number of plants analyzed for flowering time. Statistical analysis by ANOVA with posthoc Tukey HSD testing; different letters denote significant differences. **b**, Schemes of the *FT* locus including its 5.7 kb promoter and 2 kb downstream region, with Blocks A, B, C, and E indicated. A snapshot of the PIF7 ChIP-seq data around the *FT* locus is also shown, which is available at

(http://neomorph.salk.edu/aj2/pages/hchen/) [4]. **c**, *FT* transcript accumulation was measured by RT-qPCR in the indicated genotypes. Samples collected at ZT4 from 12-day old seedlings grown in long days (LD) with white light (WL) and from seedlings grown 7 days in LD (WL) followed by 5 days with additional far-red light (WL+FR). The red-to-far-red light ratio was 3.3 under WL and 0.27 under WL+FR. Relative expression was calculated using the $2^{\Delta\Delta Cq}$ method with *PP2A* as reference. Error bars show standard error of the mean (SEM) of three biological replicates. The green line shows the ratio of *FT* transcript accumulation between WL+FR and WL for each genotype, with SEM calculated from ratios across three biological replicates per condition. Statistical analysis by Welch's *t*-test, significant differences indicated by stars (ns $p>0.05$, * $p<0.05$, ** $p<0.01$, *** $p<0.001$, **** $p<0.0001$). Symbols (or ns) above bars or the line denote significance relative to WT, with colors indicating the corresponding conditions. Bracketed ns symbols indicate comparisons among Block E mutants, with colors specifying conditions.

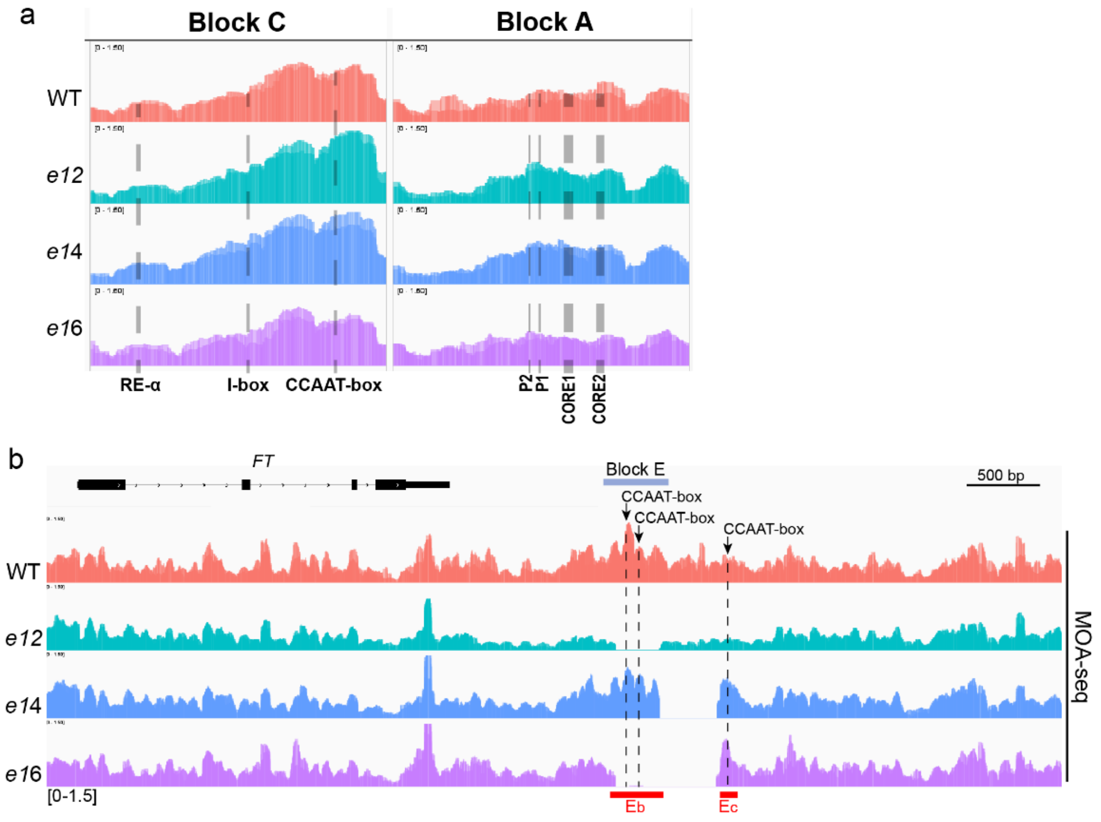

Fig. S5. MOA-seq reveals selective CCAAT-box occupancy changes around Block E in deletion mutants. a, Overlay of MOA-seq read coverage from two biological replicates at Block C (346 bp) and Block A (523 bp) in wild type (WT; red), *e12* (cyan), *e14* (blue), and *e16* (purple). The positions of conserved DNA motifs within Block C and Block A, including the conserved CCAAT-box in Block C, are indicated by dashed lines across the tracks. **b**, Overlay of MOA-seq read coverage from two biological replicates across the *FT* gene and downstream region in WT, *e12*, *e14*, and *e16*. The *FT* gene structure is shown above, with Block E indicated by a light-blue line. The two CCAAT-box motifs within Block E and the downstream CCAAT-box are marked by arrows and dashed lines.

References

1. Higo K, Ugawa Y, Iwamoto M, Korenaga T. Plant cis-acting regulatory DNA elements (PLACE) database: 1999. Nucleic Acids Res. 1999;27(1):297-300.

2. Du SS, Li L, Li L, Wei X, Xu F, Xu P, et al. Photoexcited Cryptochrome2 Interacts Directly with TOE1 and TOE2 in Flowering Regulation. Plant Physiol. 2020;184(1):487-505.

3. Zicola J, Liu L, Tanzler P, Turck F. Targeted DNA methylation represses two enhancers of FLOWERING LOCUS T in Arabidopsis thaliana. Nat Plants. 2019;5(3):300-7.

4. Willige BC, Zander M, Yoo CY, Phan A, Garza RM, Wanamaker SA, et al. PHYTOCHROME-INTERACTING FACTORs trigger environmentally responsive chromatin dynamics in plants. Nat Genet. 2021;53(7):955-61.
